# Do the Ecoregions Support Distinct Hilly and Mountain Stream Chironomid Assemblages in South-East Europe?

**DOI:** 10.3390/insects17010096

**Published:** 2026-01-14

**Authors:** Viktorija Ergović, Predrag Simović, Miran Koh, Djuradj Milošević, Dubravka Čerba, Ana Petrović, Zlatko Mihaljević

**Affiliations:** 1 Department of Biology, University of Osijek, Cara Hadrijana 8/a, 31000 Osijek, Croatia; miran.koh@biologija.unios.hr; 2 Department of Biology and Ecology, Faculty of Science, University of Kragujevac, Radoja Domanovića 12, 34000 Kragujevac, Serbia; predrag.simovic@pmf.kg.ac.rs (P.S.); ana.petrovic@pmf.kg.ac.rs (A.P.); 3 Department of Biology and Ecology, University of Niš, 18000 Niš, Serbia; djuradj.milosevic@pmf.edu.rs; 4 Department of Aquatic Ecosystems Assessment, National Water Reference Laboratory of Slovakia, Water Research Institute, Nábr. arm. gen. L. Svobodu 5, 81249 Bratislava, Slovakia; dubravka.cerba@vuvh.sk; 5 Department of Biology, University of Zagreb Faculty of Science, Horvatovac 102a, 10000 Zagreb, Croatia; zlatko.mihaljevic@biol.pmf.unizg.hr

**Keywords:** Diptera, freshwater habitats, Pannonian Lowland, Dinaric Western Balkans, Eastern Balkan

## Abstract

The region of South-East Europe, located in geologically and climatically diverse areas, hosts a wide range of freshwater habitats. However, comprehensive studies of macroinvertebrate communities are limited, and research on Chironomidae (Diptera) is particularly scarce. We present data on the diversity and structure of chironomid assemblages in hilly and mountainous streams across three ecoregions: the Pannonian Lowland (Ecoregion 11), the Dinaric Western Balkans (Ecoregion 5), and the Eastern Balkans (Ecoregion 7) and provide a comparative overview of their community patterns based on 130 samples. According to the CCA results and Monte Carlo permutation tests, water temperature, dissolved oxygen, conductivity, pH, and altitude were identified as statistically significant parameters influencing Chironomidae assemblages across the ecoregions, collectively explaining 72.20% of the variation. The higher diversity indices were recorded in each season in the Pannonian Lowland and the highest within-ecoregion similarity. Dissimilarity was highest between ER11 and ER7 and lowest between ER5 and ER7. These results demonstrate that the ecoregion was the strongest influence of the studied environmental variables on Chironomidae assemblages, with community patterns closely reflecting their spatial distribution across distinct ecoregional settings.

## Simple Summary

South-East Europe, with its diverse geological and climatic settings, is a freshwater biodiversity hotspot, yet studies linking ecoregional environmental conditions to macroinvertebrate assemblages remain limited. This study examines the diversity and structure of chironomid assemblages in hilly and mountainous streams across three ecoregions, the Pannonian Lowland, Dinaric Western Balkans, and Eastern Balkans, based on 130 samples. As one of the most abundant and ecologically versatile freshwater macroinvertebrate groups, Chironomidae serve as key indicators in biological monitoring and are the main focus of this study. The results showed that environmental factors such as water temperature, oxygen content, conductivity, pH, and altitude, explained most regional differences, with the assemblages in the Pannonian Lowland showing the highest diversity and most substantial inter-region similarity. Overall, the findings demonstrate that ecoregions strongly shape chironomid assemblages, producing clear spatial patterns across the study area.

## 1. Introduction

Freshwater ecosystems are vital components of the biosphere, supporting human health, economic value and science [1]. These ecosystems also play a crucial role in maintaining biodiversity, which, in the Anthropocene era, is far more threatened than in marine or terrestrial environments [2]. Within hydrobiological communities, freshwater macroinvertebrates are essential for sustaining aquatic ecosystem functioning, particularly through energy transfer, primary productivity, decomposition, and translocation of materials [3].

It is well known that patterns of macroinvertebrates in aquatic ecosystems are determined by the combined influence of environmental factors operating at different spatial scales [4]. Fauna in different geographical regions are shaped by past biogeographical events, geology, climate, and vegetation, while the ecological niches of individual species are determined by local abiotic conditions and biotic interactions [5]. Biogeographical realms and ecoregions have become key frameworks for understanding the organization of biodiversity, forming the basis for science-based conservation planning to protect distinct regions, habitat types, and taxa [6]. However, an important question often arises as to whether ecoregion borders truly represent meaningful delineations between distinct biotic communities [7,8].

Although the past decade has improved our understanding of aquatic macroinvertebrates in South-East Europe, several groups, including Chironomidae (Insecta, Diptera), and their connection to regional traits, remain poorly studied. Chironomidae are widely recognized as one of the most abundant and diverse groups in freshwater habitats, often making up to 50% of the total number and biomass [9] of aquatic macroinvertebrates. As a cosmopolitan group, they occur in all zoogeographical regions of the world, including Antarctica [10,11]. Their broad ecological tolerances enable species to colonize environments ranging from pristine to heavily impacted conditions [12,13,14,15]. Due to their wide range of ecological traits and sensitivity to environmental change, chironomids are considered key indicators in biological monitoring and ecosystem assessment programs [16,17,18]. However, despite their importance, this group remains understudied in many regions because of their small size, persistent taxonomic challenges, especially difficulties in achieving species-level identification, high natural variability, and a shortage of experienced specialists [19,20].

Due to its exceptionally complex hydrological network, intensive karstification, and wide variety of karst water bodies, the Dinaric Western Balkans is considered a European hotspot of aquatic biodiversity [21,22]. Accordingly, based on available research, this ecoregion is the best-studied area for Chironomidae assemblages, primarily in Croatia [15,19,23,24], with additional contributions from Montenegro [25,26] and Bosnia and Herzegovina [27], and only limited or fragmentary studies from Serbia [28,29]. In contrast, the Pannonian Lowland ecoregion has been investigated only sporadically, primarily in lentic ecosystems, with notably more research conducted in Croatia [15,30] than in Serbia [29]. However, this region in Croatia also includes the Slavonian mountains, which are characterized by diverse geological formations, including relict karst structures and distinct freshwater ecosystems [15], that remain unexplored regarding Chironomidae assemblages. The Eastern Balkan ecoregion in Serbia is represented mainly by studies of Chironomidae assemblages from the Southern Morava River basin [17,28,31]. These disparities in research effort, along with the pronounced geological and hydrological contrasts among the ecoregions, highlight the need for broader comparative studies across hill-mountain river systems, which represent some of the most ecologically sensitive freshwater habitats in these ecoregions.

The primary focus of this research was to: (i) identify Chironomidae diversity across three ecoregions of South-East Europe; (ii) assess differences in assemblage compositions among ecoregions and identify the representative taxa that contribute the most to inter-ecoregion dissimilarities in assemblages; and (iii) link assemblage patterns to the main environmental parameters influencing species distribution and ecological preferences between ecoregions.

We propose that ecoregions are the primary drivers of variation in riverine environmental conditions, such as differences in geology, hydromorphology, flow regime altitude, water quality, and substrate composition. These region-specific factors collectively shape the structure of Chironomidae assemblages. Consequently, assemblages in different ecoregions are expected to show clear ecological differentiation that reflects both local habitat properties and broader biogeographical patterns, making them indicators of the environmental conditions characteristic of each ecoregion.

## 2. Materials and Methods

### 2.1. Study Area

**Figure 1:**
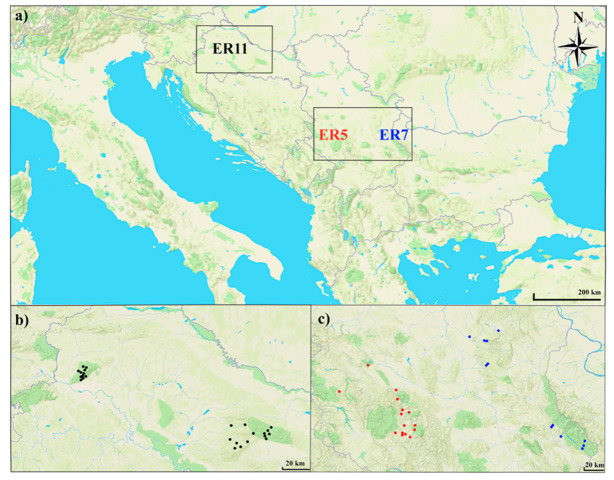
Location of sampling sites in different ecoregions: (**a**) South-East Europe with sampling ecoregion locations; (**b**) sampling sites in Croatia—black dots—Pannonian Lowland ecoregion; (**c**) sampling sites in Serbia: red dots—Dinaric Western Balkan ecoregion, blue dots—Eastern Balkan ecoregion.

The study areas are located in three distinct ecoregions of South-East Europe: Ecoregion 5–Dinaric Western Balkan (ER5, Serbia), Ecoregion 7–Eastern Balkan (ER7, Serbia) and Ecoregion 11–Pannonian Lowland (Hungarian Lowland) (ER11, Croatia) [32,33]. Hydrological surveys were conducted at 53 sites (Figure 1). The sampling sites were selected to represent typical hill-mountain, flowing-water habitats, and sampling was conducted in three seasons (spring, summer, and autumn) from 2020 to 2022.

This study included hilly and mountain streams. Streams located between 200 and 500 m a.s.l. were classified as hilly streams, whereas those situated above 500 m a.s.l. were categorized as mountain streams [34]. In the Dinaric Western Balkan ecoregion in Serbia, the study included 15 streams across different subregions: ten are located within the Ibar River valley and constitute tributaries draining into the main course of the Ibar River, one on Ovčar Mountain, three from higher-elevation sites within the National Park Kopaonik mountain massif and one from Zlatibor Mountain (Table 1), resulting in 31 samples collected. In the Eastern Balkan ecoregion, the study area covered three mountain systems: Stara Planina Mountain (six streams), Kučaj (one stream), and Beljanica Mountain (four streams), for a total of 23 samples (Figure 1).

In the Pannonian Lowland ecoregion, we sampled streams on three mountains: Medvednica, Papuk and Psunj. Parts of Papuk Mountain and Medvednica are designated as nature parks because of their unique geological, biological, and hydrological features [35,36]. Our focus was on mountain streams in the areas of Medvednica (seven streams), Papuk (five streams) and Psunj (three streams). Each stream was sampled at two different sites. A total of 76 samples were collected from the Pannonian Lowland ecoregion (Figure 1).

The number of samples varied among ecoregions due to the fact that the sampling campaigns were conducted within different projects, which led to differences in sampling efforts among ecoregions.

The characteristics of the investigated rivers and streams across the three ecoregions, including their geographic coordinates and altitudes, are presented in Table 1.

### 2.2. Sampling Procedure

Sampling was conducted in each ecoregion according to AQEM methodology [37]. Chironomidae larvae and pupae were isolated and identified from macroinvertebrate composite samples collected with a benthos hand net (mesh size 500 µm, frame size 0.25 × 0.25 m) and preserved in 96% ethanol. At each location, the dominant substrate type was sampled and all individuals were isolated and identified to the lowest possible taxonomic level using identification keys [38,39,40,41,42,43,44]. For isolation and identification of Chironomidae larvae and pupae, a stereomicroscope SMZ-171 (Motic, Hong Kong) and a microscope BA310 (Motic, Hong Kong) were used.

### 2.3. Environmental Parameters

Basic environmental parameters were measured using a handheld probe model HACH HQ30d flexi (Hach, Düsseldorf, Germany). All physical and chemical water parameters were measured in situ. Physical parameters included water temperature (°C), oxygen saturation (%), dissolved oxygen concentration (mg/L), water conductivity (µS/cm), and pH. The chemical parameter of water hardness was determined using 0.1 M HCl and calculated according to Boyd et al. [45].

### 2.4. Statistical Analysis

For all data analyses, Primer 6.0 [46] and Canoco 4.5 [47] were used. Prior to analysis, both environmental and species abundance matrices were log-transformed (log(x + 1)), and environmental variables were also normalized [46].

The total abundance of identified Chironomidae individuals was standardized as individuals per square meter for comparison across different ecoregions. Dominant taxa were identified as those comprising 5% or more of the total abundance in each ecoregion. The Shannon–Wiener (H’) [48] and Simpson’s (D) [49] diversity indices were calculated for each site using Primer 6.0 software [46]. The indices mentioned were calculated for all identified taxa in each ecoregion, including those identified at the group or genus level.

To construct the resemblance matrices, we used Bray–Curtis similarity for species abundance data and Euclidean distance for environmental variables. The Bray–Curtis measure incorporates species abundances, avoids artificial similarities, and effectively emphasizes differences among communities. Euclidean distance captures straight-line differences, retaining absolute variation needed to identify clear environmental gradients [46].

SIMPER (Similarity Percentage Partition) analyses were performed to evaluate the contribution of each taxon to the average similarity in community composition within ecoregion groups, and ANOSIM (Analysis of Similarities) analyses were used to assess differences between ecoregion groups. SIMPER identifies the contribution of individual taxa to observed differences among groups using the Bray–Curtis dissimilarity measure [46,50]. ANOSIM is a non-parametric test based on the rank distances among sample units, aiming to identify group differences between ecoregions [50].

To identify the most correlated environmental variables and interpret patterns in species assemblages, we performed a BEST (Bio-Environmental Similarity Tool) analysis using Spearman’s rank correlations [51]. Principal Component Analysis (PCA) was applied to summarize the abundance and environmental data, allowing identification of the main gradients driving variation among sites (samples) and providing a clear visualization of their multivariate relationships [46].

The influence of environmental parameters on Chironomidae assemblages (constrained analysis) was tested using a Monte Carlo permutation test (999 permutations), and the statistically significant factors were presented through Canonical-correspondence analysis (CCA) in CANOCO 4.5 software [47].

## 3. Results

### 3.1. Chironomidae Assemblages and Diversity

A total of 182 taxa, including 94 species from 130 samples, were identified across all three ecoregions. The greatest diversity of Chironomidae were collected from ER11 (143 taxa, including 66 species), followed by ER5 (64 taxa, with 41 species), and ER7 (48 taxa, with 29 species) (Supplement Table S1).

The dominant Chironomidae taxa in ER5 were: *Parametriocnemus stylatus* (Kieffer, 1924) (16.04%), *Micropsectra* sp. (9.41%), *Polypedilum convictum* (Walker, 1856) (8.83%), *Eukiefferiella brevicalcar* Kieffer, 1911 (7.81%), *Tvetenia calvescens* (Edwards, 1929) (6.99%) and *Conchapelopia* agg. (5.91%). In ER7, the most dominant taxa were: *Orthocladius thienemanni* agg. (20.08%), *Eukiefferiella fittkaui* Lehmann, 1972 (15.38%), *Orthocladius* sp. (8.72%), *Conchapelopia* agg. (8.08%), *T. calvescens* (6,68%) and *Tvetenia discoloripes* (Goetghebuer & Thienemann, 1936) (5.99%). In ER11, taxa with dominance levels above 5% of total Chironomidae abundance were: *T. calvesccens* (12.58%), *Heleniella ornaticollis* (Edwards, 1929) (7.92%), *P. stylatus* (6.53%), *Stempellinella brevis* (Edwards, 1929) (6.19%), *Brillia bifida* (Kieffer, 1909) (5.51%) and *Epoicocladius ephemerae* (Kieffer, 1924) (5.11%).

Species found in all three ecoregions were: *Potthastia longimanus* Kieffer, 1922, *Prodiamesa olivacea* (Meigen, 1818), *B. bifida*, *E. ephemerae*, *Orthocladius frigidus* (Zetterstedt, 1838), *P. stylatus*, *Rheocricotopus effusus* (Walker, 1856), *R. fuscipes* (Kieffer, 1909), *T. calvescens*, *T. discoloripes*, *Polypedilum convictum* and *Microtendipes pedellus* (De Geer, 1776).

The highest diversity was estimated for ER11, while the lowest was estimated for ER7 (Table 2).

The results of SIMPER analysis show the average similarity between samples within each ecoregion and the species contributing to similarity within each ecoregion. In ER11, the Chironomidae community has the highest similarity percentage at 37.33%, followed by ER5 and ER7 at 22.42% and 20.64%, respectively. The species that contribute most to similarity are also the most abundant in each ecoregion (Table 3).

The results of ANOSIM test suggest that the greatest dissimilarities were between groups ER11 and ER7 (R = 0.835), followed by ER11 and ER5 (R = 0.744), with the lowest dissimilarity between ER5 and ER7 (R = 0.126) (Table 4).

### 3.2. Correlation of Chironomidae Assemblages with Environmental Parameters

The SAM stream had the highest recorded altitude (1417 m a.s.l.), followed by the GOB stream (1110 m a.s.l.), both situated on Kopaonik Mountain within the ER5. The lowest altitude site was Kovačica stream sampled at 216 m a.s.l. on Papuk Mountain (ER11). Water temperature was lowest in spring and highest in summer, with the greatest variability in ER11 (7.6 °C in spring and 17.9 °C in summer).

**Figure 2:**
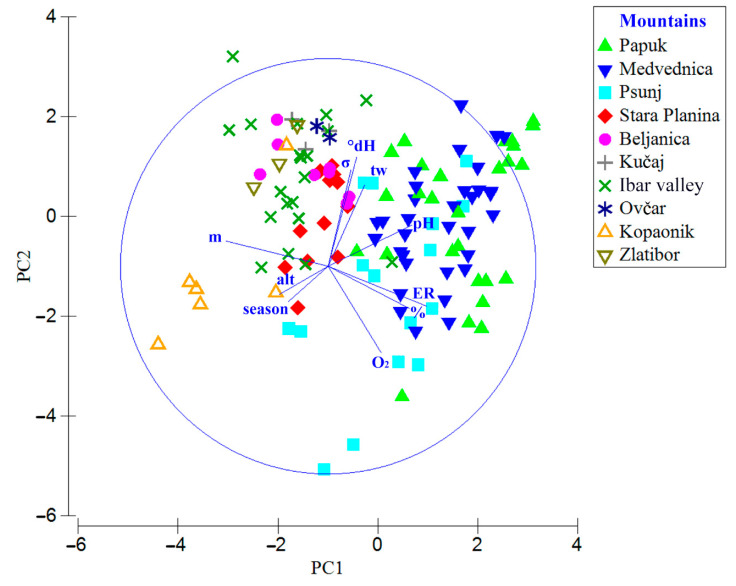
PCA plot showing variation in environmental variables across sampling sites on each mountain/subregion from all three sampled ecoregions. Abbreviations for environmental variables: tw–water temperature (°C), m—sampled mountain, alt—altitude (m a.s.l.), *σ*—conductivity, O_2_—oxygen concentration (mg/L), %—dissolved oxygen, °dH—water hardness, ER—ecoregion, season–sampling season.

Oxygen content levels ranged between 10 and 13 mg/L, and average saturation varied from 94.30% in autumn (ER11) to 117.40% in summer (ER11); in ER5 and ER7, it was around 100% in all sampling seasons. The highest average conductivity was recorded in summer in ER7 at 460 µS/cm, while the lowest was in spring in ER11 at 288 µS/cm. Water hardness depended on the geomorphological features of the mountain, with the lowest values recorded on Psunj Mountain (ER11) and Kopaonik (ER5), and the highest on Medvednica (ER11) (Figure 2, Table 5).

As a result of the BEST analysis, the Spearman correlation coefficient for Chironomidae abundance was 0.56 (*p* < 0.005), the highest among the environmental parameters, indicating that the environmental gradients specifying the ecoregion explain a significant portion of the variability in Chironomidae assemblages.

**Figure 3:**
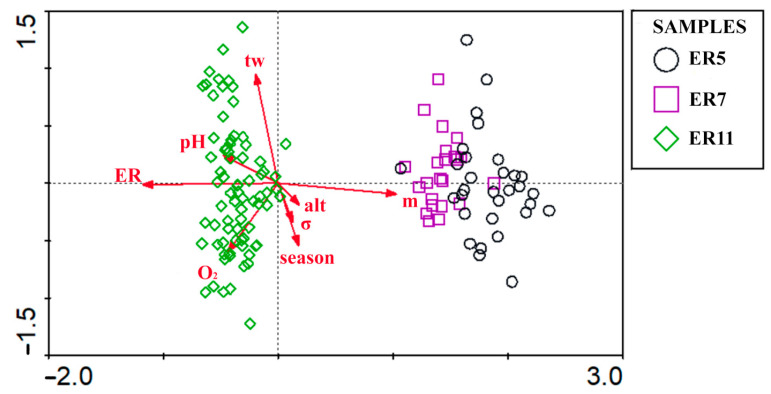
CCA plot of statistically significant environmental parameters tested in three ecoregions on Chironomidae assemblages. Red arrows indicate environmental variables: tw—water temperature (°C), m—sampled mountain, alt—altitude (m a.s.l.), *σ*—conductivity, O_2_—oxygen concentration (mg/L), ER—ecoregion, season—sampling season.

Based on CCA results and Monte Carlo permutation tests, all tested environmental parameters except water hardness and oxygen saturation levels were statistically significant for Chironomidae assemblages in ecoregions (Figure 3) and together explain 72.20% of the variation in Chironomidae assemblages. This proportion represents the constrained variance explained by the environmental variables included in the CCA model.

## 4. Discussion

European ecoregions provide a geographic framework for linking macroinvertebrate assemblages to environmental gradients [32,33], incorporating in-stream environmental conditions, and demonstrating that each ecoregion’s characteristics are major drivers of community change [52,53].

Mountain streams are generally studied in terms of changes in abiotic factors such as temperature, oxygen content, and hydrological conditions, which are associated with variations in altitude [54,55]. In the interpretation of ecological influences on species distributions, environmental factors remain the main drivers of distribution patterns within regions [56]. In this study, environmental conditions such as oxygen content, altitude, and conductivity significantly affected the Chironomidae community, and nearly all environmental parameters studied were linked to differences among ecoregions. Consistent with our hypotheses, the ecoregion emerged as the primary driver shaping Chironomidae assemblages in mountain streams, indicating that region-specific factors collectively shape Chironomidae assemblages. These findings are consistent with previous studies using comparable approaches, which have similarly highlighted the strong effect of physicochemical gradients and regional landscape characteristics on Chironomidae assemblages [24,57,58] as well as other stream macroinvertebrates [59,60].

Our results indicate that seasonal variation can represent an important predictor of aquatic macroinvertebrate community structure, which aligns with previous findings from karst ecosystems in Croatia [15,61,62]. According to these studies, seasonal changes may affect species composition due to shifts in resource availability, hydrological dynamics, or biological interactions. Conversely, Simović et al. [59] found no significant seasonal influence on macroinvertebrate communities in karst regions of the Dinaric and Eastern Balkans, attributing this to the stable environmental conditions typical of karst ecosystems. Although seasonal variation is detectable here, its effect is secondary to spatial and ecoregional drivers. We considered a more detailed exploration of seasonal mechanisms as an important direction for future research, particularly in relation to disentangling seasonal influence on Chironomidae assemblages.

From 130 samples and 184 taxa, more than half of the taxa were found in ER11, along with higher diversity indices, likely due to the sampling effort in the ER11 mountain streams. A larger sampling effort shows that macroinvertebrate richness increases systematically with the number of replicate samples, and that insufficient sampling effort underestimates biodiversity and alters assessment results [63,64]. Alternatively, this may be explained by spatial connectivity between sampled localities and the limited dispersal ability of Chironomidae species [18,65]. The idea that colonization was easier at nearby locations is also supported by SIMPER analysis results, which showed greater similarity within ER11 than within the other two ecoregions. Species richness was relatively low in ER5 and ER7.

The positive correlation between ecoregion with Chironomidae assemblages abundance indicates that geographical distance has influenced their structure. The greatest dissimilarity was observed between the two most distant localities within ER11 and ER7. Results from ANOSIM analysis suggested very strong separation and highly distinct assemblages. According to Dorić et al. [24], in a study of lakes in ER5 and ER11, Chironomidae species composition was found to be more similar among geographically closer lakes, which corresponds with our results. Chironomidae assemblages in ER7 and ER5, as indicated by ANOSIM results, showed weak separation and considerable overlap. The results of higher similarity between ER5 and ER7 can be explained by their comparable river types, which are predominantly represented by medium-sized streams and rivers characterized by similar hydromorphological features, fast-flowing conditions, and coarse substrate composition. Furthermore, all investigated sites in both ecoregions belong to the Morava River basin, which encompasses watercourses that support diverse fish and macroinvertebrate assemblages [59,66]. In contrast, ER11 represents a hydromorphologically distinct system, being largely dominated by smaller mountainous streams, which contributes to the observed differences in community composition [67].

Accurate identification of Chironomidae species is a valuable tool for ecological assessments because they inhabit a wide range of microhabitats and display high species richness and abundance in nearly all freshwater ecosystems [9]. Along with spatial connectivity, environmental gradients related to species-specific conditions [33] can provide additional information on community structuring. The dominant Chironomidae species in this study are found in running water, some of which are also considered cosmopolitan, such as *Parametriocnemus stylatus*, *Brillia bifida* and *Polypedilum convictum*. These species inhabit streams of all types and forms. When found in lakes, these individuals were transported by the brooks from which they originated [38,39]. The presence of Diamesinae and the dominance of Orthocladiinae taxa were favorable for mountain stream ecosystems across all ecoregions [68,69]. The species in this study are considered indicator species of good water quality and are used in biomonitoring research [16,17,18]. In addition to the dominant species, higher-elevation sites and fast-flowing streams also hosted Diamesinae (*Diamesa insignipes* Kieffer, 1908; *Diamesa tonsa* (Haliday, 1856); *Diamesa zernyi* Edwards, 1933 and *Diamesa permacra* (Walker, 1856)). Alongside with other Diamesinae, *Boreoheptagyia monticola* was present in the Ibar River valley. The larvae of *B. monticola* inhabit the lowest parts of waterfalls, in rheomadicolous areas where water constantly rains down on stones [39,70]. The high diversity of Chironomidae in the mountain streams of the studied mountains was consistent with other studies [68,69,71], with high substrate heterogeneity [72] shaped by ecoregions as the primary drivers of variation in stream environmental conditions [33]. Future research may build on the results of this study by including additional environmental variables that could provide further information about habitat and stream morphological characteristics, such as stream width, size, stream order, and hydrological factors like stream discharge. These variables could influence the specific conditions of the ecoregion and thereby improve our understanding of the formation of Chironomidae assemblages.

## 5. Conclusions

Among the environmental parameters studied, altitude, water temperature and oxygen content were the most important drivers of region-specific factors shaping the structure of the Chironomidae assemblages. Due to their high taxonomic variability, it is essential to identify Chironomidae to the species level, as their abundance can influence community structure investigations and is often overlooked. Our findings show that Chironomidae assemblages are most strongly influenced by ecoregion, with patterns closely reflecting their spatial distribution across multiple ecoregions. Ecoregions reflect influences beyond the environmental parameters measured in this study, which may explain their prominence as the most influential variable. The observed patterns emphasize the value of ecoregions as informative frameworks for interpreting ecological variability across freshwater systems, particularly in insufficiently explored regions of South-East Europe, thereby contributing new ecological knowledge on the distribution and structure of Chironomidae assemblages in this area.

## Figures and Tables

**Table 1:** Characteristics of the investigated sampling sites across the three ecoregions, geographical coordinates are expressed in the decimal degrees WGS 84 map.

Ecoregion	Subregion/Mountain	Sampling Sites		Latitude	Longitude	Altitude (m a.s.l.)
		Name	Abbreviation			
Dinaric Western Balkan	Ibar River valley	Raška	RAS	43.276880	20.612114	397
		Trnavska	TRN1	43.292230	20.525566	470
			TRN2	43.284657	20.613573	402
		Brevnica	BRV	43.357304	20.633174	424
		Bresnička	BRE	43.588607	20.548409	456
		Gokčanica	GOK	43.473032	20.691855	429
		Pusto Polje	PUS	43.493673	20.613435	340
		Badanj	BAD	43.279844	20.646658	429
		Studenica	STU	43.458620	20.599604	347
		Lopatnica	LOP	43.670457	20.531275	282
	Zlatibor	Gostiljska	GOS	43.657739	19.839147	731
	Ovčar	Banjski Stream	BAN	43.889694	20.188167	328
	Kopaonik	Gobeljska	GOB	43.359660	20.767307	1110
		Samokovska	SAM	43.320554	20.760525	1417
		Rudnička	RUD	43.254548	20.701102	555
Eastern Balkan	Stara Planina	Jelovičko Vrelo	JEL	43.183403	22.833464	791
		Jelovička River	JELR	43.221865	22.843696	780
		Visočica	VIS	43.157170	22.815234	705
		Temštica	TEM	43.263178	22.550675	620
		Bigar Stream	BIG	43.350666	22.440118	537
		Stanjanska	STA	43.354482	22.444195	458
	Beljanica	Milivska	MIL	44.136619	21.435479	713
		Mlava Spring	MLA	44.191692	21.783778	315
		Malo Vrelo	MVR	44.104851	21.615317	324
		Lisine	LIS1	44.100783	21.639465	402
	Kučaj	Grza	GRZ1	43.898722	21.649778	430
			GRZ2	43.890014	21.639456	382
Pannonian Lowland	Papuk	Dubočanka	DUB1	45.498067	17.685110	510
			DUB2	45.468072	17.657140	313
		Veličanka	VEL1	45.499701	17.644420	540
			VEL2	45.486672	17.647688	350
		Kovačica	KOV1	45.521167	17.673853	587
			KOV2	45.542953	17.703529	216
		Bijela	BIJ1	45.560443	17.461190	567
			BIJ2	45.555471	17.331388	273
		Brzaja	BRZ1	45.502344	17.539913	326
	Psunj	Brzaja	BRZ2	45.448822	17.483030	241
		Sivornica	SIV1	45.404831	17.363988	765
			SIV2	45.464565	17.321802	334
		Cikotska	CIK1	45.414082	17.419074	725
			CIK2	45.440068	17.381455	366
	Medvednica	Kraljevec	KRALJ1	45.881918	15.942720	589
			KRALJ2	45.865689	15.948320	384
		Bliznec	BLIZ1	45.896938	15.957089	819
			BLIZ2	45.878832	15.976946	402
		Bistra	BIS2	45.906222	15.930406	519
			BIS3	45.912720	15.912566	320
			BIS1	45.920034	15.965598	694
		Mali Potok	MP1	45.876573	15.937076	593
			MP2	45.857923	15.936079	310
		Rakova Noga	RN	45.934875	15.980445	534
		Vidak	VID	45.941839	15.954094	315
		Veliki Potok	VP	45.858266	15.934939	301

**Table 2:** The calculated average of diversity indices in three sampled seasons.

	Spring		Summer		Autumn	
Ecoregion/Diversity	Shannon–Wiener	Simpson	Shannon–Wiener	Simpson	Shannon–Wiener	Simpson
ER5	1.67	0.75	1.54	0.72	1.34	0.71
ER7	1.50	0.68	1.73	0.80	1.45	0.71
ER11	2.28	0.83	2.13	0.79	2.01	0.78

**Table 3:** Taxa contributing more than 5% to within-ecoregion similarity based on SIMPER analysis.

	E11	E7	E5
Average Similarity (%)	37.33	22.42	20.64
Taxa	taxa contribution (%)		
*Parametriocnemus stylatus*	12.69	12.29	30.52
*Heleniella ornaticollis*	11.70		
*Tvetenia calvescens*	10.67	18.27	
*Conchapelopia* agg.	8.35	15.61	8.82
*Corynoneura* sp.	8.25		
*Brillia bifida*	5.76		7.76
*Eukiefferiella fittkaui*		10.25	
*Orthocladius thienemanni* agg.		9.25	
*Orthocladius* sp.		6.33	
*Micropsectra* sp.		10.55	20.29
*Tvetenia discoloripes*			8.69
*Polypedilum convictum*			5.59

**Table 4:** R values for the ANOSIM test results among the tested ecoregion groups with statistical significance levels.

Ecoregion Pairs	R-Values	*p*-Value
ER11 and ER7	0.835	<0.005
ER11 and ER5	0.744	<0.005
ER7 and ER5	0.126	<0.005

**Table 5:** Average values of measured environmental parameters by season and ecoregion.

Ecoregion	Season	Water Temperature (°C)	Dissolved Oxygen Concentration (mg/L)	Oxygen Saturation (%)	Water Conductivity (µS/cm)	pH Values	Water Hardness Values (°dH)
ER5	spring	9.0	11.0	100.60	362	7.8	10.0
	summer	13.1	9.9	100.60	306	7.8	8.6
	autumn	9.7	10.8	102.50	311	7.8	8.5
ER7	spring	10.1	10.1	99.10	353	7.9	9.7
	summer	10.1	10.2	100.30	460	7.2	12.7
	autumn	10.9	10.1	98.90	348	7.7	9.6
ER11	spring	7.6	13.6	117.40	288	8.0	7.0
	summer	17.9	10.6	116.80	334	8.6	9.8
	autumn	9.5	10.4	94.30	316	8.3	9.1

## Data Availability

The original contributions presented in this study are included in the article/Supplementary Material. Further inquiries can be directed to the corresponding author.

## Supplementary Materials

The following supporting information can be downloaded at: https://www.mdpi.com/article/10.3390/insects17010096/s1, Table S1. Chironomidae taxa list identified in investigated ecoregions. ER5-Dinaric Western Balkan, ER7-Eastern Balkan, ER11-Pannonian Lowland.
