# Supplementary material for: Do the Ecoregions Support Distinct Hilly and Mountain Stream Chironomid Assemblages in South-East Europe?
Source: Insects. 2026 Jan 14;17(1):96. doi: 10.3390/insects17010096 (PMC12841726; doi:10.3390/insects17010096)
Supplement: Supplementary file 1 [file insects-17-00096-s001.zip › insects-4046716-supplementary.pdf]

Table S1. Chironomidae taxa list identified in investigated ecoregions. ER5-Dinaric Western Balkan, ER7-Eastern Balkan, ER11-Pannonian Lowland.

| Taxa | Ecoregion |
|---|---|
| *Ablabesmyia longistyla* Fittkau, 1962 | ER5 |
| *Apsectrotanypus* sp. | ER11 |
| *Apsectrotanypus trifascipennis* (Zetterstedt, 1838) | ER11 |
| *Clinotanypus nervosus* (Meigen, 1818) | ER11 |
| *Conchapelopia* agg. | ER5, ER7, ER11 |
| *Krenopelopia* sp. | ER11 |
| *Larsia* sp. | ER11 |
| *Macropelopia nebulosa* (Meigen, 1804) | ER5, ER7 |
| *Macropelopia* sp. | ER5, ER7, ER11 |
| *Natarsia* sp. | ER11 |
| *Nilotanypus dubius* (Meigen, 1804) | ER7, ER11 |
| *Paramerina* sp. | ER11 |
| *Pentaneurella* sp. | ER11 |
| *Procladius* sp. | ER7 |
| *Psectrotanypus varius* (Fabricius, 1787) | ER5 |
| *Schineriella* sp. | ER11 |
| Tanypodinae Gen sp. | ER5, ER11 |
| *Trissopelopia* sp. | ER5 |
| *Xenopelopia* sp. | ER11 |
| *Zavrelimyia* sp. | ER5, ER11 |
| *Boreoheptagyia monticola* (Serra-Tosio, 1964) | ER5 |
| *Diamesa insignipes* Kieffer, 1908 | ER5, ER7 |
| *Diamesa permacra* (Walker, 1856) | ER11 |
| *Diamesa* sp. | ER7, ER11 |
| *Diamesa tonsa* (Haliday, 1856) | ER5, ER7 |
| *Diamesa zernyi* Edwards, 1933 | ER7 |
| Diamesinae Gen sp. | ER11 |
| *Monodiamesa bathyphila* (Kieffer, 1918) | ER11 |
| *Paraboreochlus minutissimus* (Strobl, 1894) | ER11 |
| *Potthastia longimanus* Kieffer, 1922 | ER5, ER7, ER11 |
| *Potthastia gaedii* (Meigen, 1838) | ER7, ER11 |
| *Potthastia* sp. | ER7 |
| *Prodiamesa olivacea* (Meigen, 1818) | ER5, ER7, ER11 |
| *Brillia bifida* (Kieffer, 1909) | ER5, ER7, ER11 |
| *Brillia flavifrons* (Johannsen, 1905) | ER7 |
| *Brillia longifurca* Kieffer, 1921 | ER11 |
| *Brillia* sp. | ER11 |
| *Chaetocladius dentiforceps* gr. | ER5 |
| *Chaetocladius* sp. | ER11 |
| *Corynoneura celeripes* Winnertz, 1852 | ER11 |
| *Corynoneura* gr. *scutellata* | ER11 |
| *Corynoneura* sp. | ER5, ER7, ER11 |
| *Cricotopus bicinctus* (Meigen, 1818) | ER5, ER7 |
| *Cricotopus curtus* Hirvenoja, 1973 | ER5 |
| *Cricotopus* sp. | ER7, ER11 |
| *Cricotopus tremulus* (Linnaeus, 1758) | ER5, ER11 |

| | |
|---|---|
| *Cricotopus triannulatus* agg. | ER11 |
| *Cricotopus* sp./*Orthocladius* sp. | ER11 |
| *Epoicocladius* sp. | ER5 |
| *Epoicocladius ephemerae* (Kieffer, 1924) | ER5, ER7, ER11 |
| *Eukiefferiella fittkaui* Lehmann, 1972 /*minor* Edwards, 1929 | ER11 |
| *Eukiefferiella lobifera* Goetghebuer, 1934 | ER5, ER11 |
| *Eukiefferiella brevicalcar* Kieffer, 1911 | ER7, ER11 |
| *Eukiefferiella claripennis* gr. | ER11 |
| *Eukiefferiella dittmari* Lehmann, 1972 | ER11 |
| *Eukiefferiella fittkaui* Lehmann, 1972 | ER5, ER7 |
| *Eukiefferiella brehmi* gr. | ER5 |
| *Eukiefferiella devonica* gr. | ER5, ER7, ER11 |
| *Eukiefferiella gracei* Edwards, 1929 | ER11 |
| *Eukiefferiella similis* Goetghebuer, 1939 | ER11 |
| *Eukiefferiella* sp. | ER5, ER7, ER11 |
| *Eukiefferiella tirolensis* Goetghebuer, 1938 | ER11 |
| *Eukiefferiella tirolensis* Goetghebuer, 1938/ *brevicalcar* (Kieffer, 1911) | ER11 |
| *Georthocladius* sp. | ER11 |
| *Gymnometriocnemus* sp./*Bryophaenocladius* sp. | ER11 |
| *Heleniella ornaticollis* (Edwards, 1929) | ER11 |
| *Heterotrissocladius marcidus* (Walker, 1856) | ER11 |
| *Krenosmittia halvorseni* (Cranston & Saether, 1986) | ER11 |
| *Krenosmittia* sp. | ER11 |
| *Lymnophyes* sp. | ER11 |
| *Metriocnemus albolineatus* (Meigen, 1818)/ *obscuripes* (Holmgren, 1869) | ER11 |
| *Metriocnemus* sp. | ER11 |
| *Nanocladius balticus* (Palmen, 1959) | ER5 |
| *Nanocladius rectinervis* (Kieffer, 1911) | ER11 |
| *Nanocladius* sp. | ER11 |
| Orthocladiinae Gen sp. | ER11 |
| *Orthocladius (Euorthocladius)* sp. | ER11 |
| *Orthocladius (Orthocladius)* sp. | ER11 |
| *Orthocladius (Symposiocladius) lignicola* Kieffer, 1914 | ER11 |
| *Orthocladius (Euorthocladius) ashei* (Soponis, 1990) | ER5, ER11 |
| *Orthocladius (Mesorthocladius) frigidus* (Zetterstedt, 1838) | ER5, ER7, ER11 |
| *Orthocladius (Euorthocladius) luteipes* Goetghebuer, 1938 | ER5 |
| *Orthocladius (Euorthocladius) rivulorum* Kieffer, 1909 | ER11 |
| *Orthocladius (Euorthocladius) saxosus* (Tokunaga, 1939) | ER5 |
| *Orthocladius* sp. | ER5, ER7, ER11 |
| *Orthocladius (Euorthocladius) thienemanni* agg. | ER5, ER7 |
| *Orthocladius (Orthocladius) wetterensis* Brundin, 1956 | ER5 |
| *Othocladius (Orthocladius) rubicundus* (Meigen, 1818) | ER5, ER11 |
| *Paracricotopus* sp. | ER11 |
| *Parakiefferiella* sp. | ER11 |
| *Parametriocnemus* sp. | ER7, ER11 |
| *Parametriocnemus stylatus* (Kieffer, 1924) | ER5, ER7, ER11 |
| *Paratrichocladius nivalis* (Goetghebuer, 1838) | ER7 |
| *Paratrichocladius rufiventris* (Meigen, 1830) | ER5, ER7 |
| *Paratrissocladius excerptus* (Walker, 1856) | ER5, ER11 |
| *Paraphaenocladius penerasus* (Edwards, 1929) | ER11 |

| | |
|---|---|
| *Paraphaenocladius pseudirritus* Strenzke, 1950 | ER11 |
| *Paraphaenocladius* sp. | ER11 |
| *Psectrocladius psilopterus* (Kieffer, 1906) | ER11 |
| *Psectrocladius* sp. | ER11 |
| *Pseudosmittia* sp. | ER11 |
| *Rheocricotopus atripes* (Kieffer, 1913) | ER11 |
| *Rheocricotopus chalybeatus* (Edwards, 1929) | ER5 |
| *Rheocricotopus effusus* (Walker, 1856) | ER5, ER7, ER11 |
| *Rheocricotopus fuscipes* (Kieffer, 1909) | ER5, ER7, ER11 |
| *Rheocricotopus glabricollis* (Meigen, 1830) | ER7 |
| *Rheocricotopus* sp. | ER11 |
| *Rheosmittia* sp. | ER11 |
| *Smittia* sp. | ER11 |
| *Symbiocladius rhithrogenae* (Zavrel, 1924) | ER11 |
| *Synorthocladius semivirens* (Kieffer, 1909) | ER5, ER11 |
| *Thienemanniella clavicornis* (Kieffer, 1911) /*vittata* (Edwards, 1924) | ER11 |
| *Thienemannia gracilis* Kieffer, 1909 | ER11 |
| *Thienemannia* sp. | ER11 |
| *Thienemannia* sp. /*Metriocnemus* sp. | ER11 |
| *Thienemanniella acuticornis* (Kieffer, 1912) | ER5, ER11 |
| *Thienemanniella majuscula* (Edwards, 1924) | ER7 |
| *Thienemanniella* sp. | ER5, ER7, ER11 |
| *Thienemanniella* sp. / *Corynoneura* sp. | ER11 |
| *Thienemanniella clavicornis* (Kieffer, 1911) | ER5 |
| *Tvetenia calvescens* (Edwards, 1929) | ER5, ER7, ER11 |
| *Tvetenia calvescens* (Edwards, 1929) / *bavarica* (Goetghebuer, 1934) | ER5 |
| *Tvetenia calvescens* (Edwards, 1929) /*discoloripes* (Goetghebuer & Thienemann, 1936) | ER11 |
| *Tvetenia discoloripes* (Goetghebuer & Thienemann, 1936) | ER5, ER7, ER11 |
| *Tvetenia discoloripes* (Goetghebuer & Thienemann, 1936) /*verralli* (Edwards, 1929) | ER5, ER11 |
| *Tvetenia* sp. | ER5, ER11 |
| *Tvetenia verralli* (Edwards, 1929) | ER5 |
| *Chironomus parathummi* Keyl, 1961 | ER11 |
| *Chironomus* sp. | ER7 |
| *Cryptochironomus obreptans* (Walker, 1856) /*supplicans* (Meigen, 1830) | ER11 |
| *Cryptochironomus* sp. | ER11 |
| *Cyphomella cornea* Saether, 1977 | ER11 |
| *Cyphomella* sp. | ER11 |
| *Demicryptochironomus vulneratus* (Zetterstedt, 1838) | ER11 |
| *Endochironomus albipennis* (Meigen, 1830) | ER11 |
| *Endochironomus* sp. | ER7, ER11 |
| *Harnischia curtilamellata* (Malloch, 1915) | ER11 |
| *Harnischia fuscimana* Kieffer, 1921 | ER11 |
| *Kloosia pusilla* (Linnaeus, 1767) | ER11 |
| *Microtendipes chloris* agg. | ER11 |
| *Microtendipes pedellus* (De Geer, 1776) | ER5, ER7, ER11 |
| *Microtendipes pedellus* (De Geer, 1776)/ *diffinis* Edwards, 1929 | ER11 |
| *Microtendipes rydalensis* (Edwards, 1929) | ER5, ER11 |
| *Microtendipes* sp. | ER11 |
| *Microtendipes tarsalis* agg. | ER5 |
| *Paracladopelma doris* gr. | ER11 |

| | |
|---|---|
| *Paracladopelma nigritulum* (Goetghebuer, 1942) | ER11 |
| *Paracladopelma* sp. | ER11 |
| *Paralauterborniella nigrohalteralis* (Malloch, 1915) | ER11 |
| *Paratendipes albimanus* (Meigen, 1818) | ER7 |
| *Paratendipes* sp. | ER11 |
| *Phaenopsectra flavipes* (Meigen, 1818) | ER11 |
| *Polypedilum acifer* Townes, 1945 | ER11 |
| *Polypedilum albicorne* (Meigen, 1838) | ER11 |
| *Polypedilum bicrenatum* Kieffer, 1921 | ER11 |
| *Polypedilum convictum* (Walker, 1856) | ER5, ER7, ER11 |
| *Polypedilum cultellatum* Goetghebuer, 1931 | ER11 |
| *Polypedilum nubeculosum* (Meigen, 1804) | ER11 |
| *Polypedilum pedestre* (Meigen, 1830) | ER11 |
| *Polypedilum scalaenum* (Schrank, 1803) | ER11 |
| *Polypedilum* sp. | ER5, ER11 |
| *Saetheria reissi* Jackson, 1977 | ER5 |
| *Saetheria* sp. | ER11 |
| *Stictochironomus pictulus* (Meigen, 1930) | ER7 |
| *Stictochironomus sticticus* agg. | ER11 |
| *Tribelos* cf. *intextum* (Walker, 1856) | ER11 |
| *Cladotanytarsus* sp. | ER11 |
| *Micropsectra bidentata* (Goetghebuer, 1921) | ER7, ER11 |
| *Micropsectra* sp. | ER5, ER7, ER11 |
| *Paratanytarsus dissimilis* agg. | ER7 |
| *Paratanytarsus* sp. | ER5, ER11 |
| *Rheotanytarsus* sp. | ER5, ER7, ER11 |
| *Stempellinella brevis* (Edwards, 1929) | ER11 |
| *Tanytarsus brundini* Lindeberg, 1963 | ER5 |
| *Tanytarsus gibbosiceps* Kieffer, 1922 | ER5 |
| *Tanytarsus heusdensis* Goetghebuer, 1923 | ER11 |
| *Tanytarsus pallidicornis* (Walker, 1856) | ER7 |
| *Tanytarsus* sp. | ER5, ER7, ER11 |
| Tanytarsini Gen sp. | ER11 |
| Chironomidae Gen sp. (larvula) | ER11 |
